# Study protocol for Early Tracking of Childhood Health determinants (ETCHED): A longitudinal observational life course study

**DOI:** 10.1186/s12889-024-20176-7

**Published:** 2024-09-29

**Authors:** Elsa Vazquez Arreola, Dean V. Coonrod, Sourav Roy Choudhury, William C. Knowler, Mary Hoskin, Dorota Wasak, Rachel Williams, Robert L. Hanson, Elena Pack, Rachel Caballero, Amanda Gonzalez, Madhumita Sinha

**Affiliations:** 1 Diabetes Epidemiology and Clinical Research Section, National Institute of Diabetes and Digestive and Kidney Diseases (NIDDK), Phoenix, AZ USA; 2 Department of Obstetrics and Gynecology, Valleywise Health Medical Center, Phoenix, AZ USA; 3 Department of Obstetrics and Gynecology, Affiliate Faculty Creighton, University School of Medicine Phoenix Regional Campus, Phoenix, AZ USA; 4 National Institutes of Health (NIH), National Institute of Diabetes and Digestive and Kidney Diseases (NIDDK), Phoenix Epidemiology and Clinical Research Branch (PECRB), 1550 E. Indian School Rd., Phoenix, AZ 85014 USA

**Keywords:** Childhood obesity, Minority health, Gestational diabetes, Youth onset diabetes

## Abstract

**Background:**

The prevalence of childhood obesity and diabetes continues to rise in the United States (US), especially among minority populations. The objective of the Early Tracking of Childhood Health Determinants (ETCHED) study is to investigate the role of adverse fetal and early-life risk exposures that contribute to the development of childhood obesity and metabolic risk.

**Methods:**

ETCHED is a longitudinal observational study of American Indian/Alaska Native (AI/AN) and Hispanic pregnant woman and their offspring. Pregnant mothers ≥ 18 years old are enrolled at a large public hospital system in the southwestern US. Enrolled mothers are followed through pregnancy, delivery, and the maternal/offspring dyad will be followed until the child’s 18th birthday. At each maternal visit, questionnaires assessing medical history, diet, physical activity, sleep, perceived stress, and socioeconomic and sociocultural information are obtained. Standard laboratory tests during maternal visits include glycemic measures, lipids, and renal function. Additional bio samples obtained include venous blood samples and cord blood for obesity/metabolic biomarkers and genetic/epigenetic testing, urinalysis, placental tissue for examining functional pathways, breast milk for metabolomics, and stool for metabolites and microbiome analysis. The offspring will have 6 infant/toddler visits at 6–12 weeks, 4 months, 6 months, 18 months, 2 and 3 years respectively. Thereafter, they will undergo comprehensive research visits (major visits) at 4–5 years, 6–9 years, 10–13 years, and 14–17 years. The major visits in children include detailed medical history, anthropometry, developmental assessment, socioeconomic and environmental assessments (food insecurity, family structure, and childcare), feeding and activity, biochemical tests, genetics/epigenetic testing, and ultrasound elastography. Electronic health records will be reviewed for additional clinical information. The primary analysis will constitute estimation of correlation coefficients between continuous variables. The planned study duration in this ongoing study is 23-years.

**Discussion:**

This is a life course study that that will examine biological and environmental risk factors for obesity and cardiometabolic risk from the intrauterine period to early childhood and adolescence in a population with high-risk of obesity and type 2 diabetes in the United States. The ETCHED study would also provide a unique opportunity to combine multi-omics and clinical data to create novel integrative models to predict the cardiometabolic risk associated with childhood obesity and possibly identify etiopathogenetic mechanisms and future targets of intervention.

**Trial registration number:**

ClinicalTrials.gov identifier: NCT03481829. Updated July 19, 2024, https://clinicaltrials.gov/study/NCT03481829?cond=ETCHED&rank=1.

## Background

The prevalence of childhood obesity and severe obesity continues to remain high and rising in the United States (US) with one-fifth of children and adolescents between the ages of 2–19 years currently having this chronic condition [1, 2]. A significant racial and ethnic disparity in obesity prevalence is also evident with children in vulnerable minority population groups, children who are of Hispanic ethnicity and/or American Indian/Alaska Native (AI/AN), bearing a major burden of disease [1–4]. With the increase in childhood obesity, there has also been a parallel and contemporaneous rise in the incidence of obesity-associated co-morbidities such as youth-onset type 2 diabetes (T2D) that was previously uncommon in the pediatric age group in the United States [5]. Based on the SEARCH for Diabetes in Youth study, a population-based registry of diabetes in youth (< 20 years) with surveillance, the incidence of clinically-diagnosed T2D has risen at a higher rate than that of type 1 diabetes (T1D) and for both types, the rates of increase have been more pronounced in minority youth particularly in Hispanic and AI/AN communities [6].

We have previously shown that in a cohort of AI/AN children living in the southwestern US, children with the most severe obesity had a 2- to 5-fold increased risk of developing diabetes by 20 years of age when compared to their peers with the least severe obesity, concluding that obesity severity is a key driver of the epidemic of youth-onset diabetes [7]. The disease course of youth with T2D is also more virulent compared to youth suffering from T1D, since they exhibit a higher risk for diabetes related micro vasculopathy [8]. Hence increased adiposity and subsequent obesity induced metabolic dysfunction manifesting early in the life-course may lead to adverse health outcomes in young adulthood including the risk of premature mortality [9].

Obesity and T2D are determined by a combination of biological and environmental factors, the most prominent of which is in utero exposure to maternal diabetes [10]. In a Southwestern US American Indian community, the offspring of mothers with diabetes have a greater risk of obesity and T2D than do offspring of fathers with diabetes or of mothers who developed diabetes after the pregnancy [11]. The increased risk of obesity and diabetes in the offspring was not entirely due to genetic susceptibility but was also attributable to the diabetic intrauterine environment [10, 11]. Intrauterine exposure to gestational diabetes was also associated with obesity in Mexican American children [12]. This phenomenon leads to a vicious cycle whereby the female offspring of a pregnant woman with diabetes during pregnancy are more likely to develop obesity and T2D before or during their child-bearing years, thus repeating the cycle across generations and leading to increasing prevalence of obesity and diabetes in youth [13, 14].

The fetal origins of adult disease theory describes the effect of adverse maternal conditions such as suboptimal nutrition during pregnancy resulting in structural and functional changes in the fetus (fetal programming). These changes predispose the offspring to cardiometabolic risk such as coronary heart disease, insulin resistance, and diabetes [15, 16]. The concept of Developmental Origins of Health and Disease (DOHaD) extends the fetal origins theory (critical period theory) and links early life biological and environmental exposures, to chronic disease risk during the life course [17].

The primary aim of the Early Tracking of Childhood Health Determinants (ETCHED) study is to examine the associations of maternal risk factors, including pre-pregnancy body mass index, gestational weight gain, and glycemia during pregnancy with offspring birth weight, length, and age/sex adjusted body mass index (BMI) percentile and z-score from birth until 2 years of age; a secondary objective includes studying these associations with BMI percentile and z-score from 2 to 18 years. Other secondary objectives study the role of maternal clinical, genetic, biochemical, lifestyle, and psychosocial factors during pregnancy in mediating the associations between maternal risk factors and birth weight of the offspring, and weight, adiposity, metabolic profile, and neurodevelopment; this during infancy, childhood, and adolescence. The ETCHED study will only enroll participants of Hispanic ethnicity and/or American Indian.

## Methods/design

### Study design

The Early Tracking of Childhood Health Determinants (ETCHED) study is a single-site longitudinal observational study being conducted by the National Institute of Diabetes and Digestive and Kidney Diseases (NIDDK), Phoenix Epidemiology and Clinical Research Branch (PECRB). Pregnant women are screened for eligibility and study enrollment began in April 2022 at the antenatal clinics at Valleywise Health Medical Center (VHMC) in Phoenix, Arizona. VHMC is the largest public hospital system in Maricopa County, Arizona, in the southwestern US.

### Eligibility criteria

Pregnant women aged 18 years or older, of AI/AN or of Hispanic race/ethnicity by self-report are eligible for participation. Participants must agree to continue with research study participation (both mother and their offspring) for at least 3-years post-delivery. Incarcerated women, women with a non-viable pregnancy, or those who are unable to consent for any reason, are excluded from participating in the study.

### Study timeline

The ETCHED study began enrolling participants in April 2022, and plans to enroll up to 750 mothers and their child (or children, if multiple gestation/pregnancy) for a total enrollment up to 1,500 persons over 5 years. The maternal/offspring dyad will be followed until the offspring’s 18th birthday. The planned study duration is 23-years with a completion date of April 2045.

The schedule of activities at research visits for both mother and child participants is shown in Table 1.

**Table 1:** Schedule of activities for ETCHED

	Weeks Gestation				Child’s Age																				
	**9–12**	**13–28**	**≥ 29**	**Delivery**	**6–12 wks**	**4 mo.**	**6 mo.**	**1 yr**	**18 mo.**	**2 yrs**	**3 yrs**	**4 yrs**	**5 yrs**	**6 yrs**	**7 yrs**	**8 yrs**	**9 yrs**	**10 yrs**	**11 yrs**	**12 yrs**	**13 yrs**	**14 yrs**	**15 yrs**	**16 yrs**	**17 yrs**
**Maternal measures**																									
Demographics	X	X	X		X			X		X		X*		X*				X*				X*			
Medical History	X	X	X		X			X		X		X*		X*				X*				X*			
Medical Record Review	X	X	X	X	X			X		X		X*		X*				X*				X*			
Labs (Blood and urine)	X	X	X		X			X		X		X*		X*				X*				X*			
OGTT					X			X		X		X*		X*				X*				X*			
DNA/RNA	X		X					X		X		X*		X*				X*				X*			
Research blood and urine for storage	X	X	X		X			X		X		X*		X*				X*				X*			
Breastmilk (as available)					X			X		X															
Stool microbiome	X		X		X			X		X		X*		X*				X*				X*			
Vitals	X	X	X		X			X		X		X*		X*				X*				X*			
Questionnaires	X	X	X		X			X		X		X*		X				X*				X*			
**Child measures**				**Birth**	**6–12 wks**	**4 mo.**	**6 mo.**	**1 yr**	**18 mo.**	**2 yrs**	**3 yrs**	**4 yrs**	**5 yrs**	**6 yrs**	**7 yrs**	**8 yrs**	**9 yrs**	**10 yrs**	**11 yrs**	**12 yrs**	**13 yrs**	**14 yrs**	**15 yrs**	**16 yrs**	**17 yrs**
Medical History					X	X	X	X	X	X	X	X	X	X	X	X	X	X	X	X	X	X	X	X	X
Medical Record Review				X	X	X	X	X	X	X	X	X	X	X	X	X	X	X	X	X	X	X	X	X	X
Vital Signs											X	X	X	X	X	X	X	X	X	X	X	X	X	X	X
Limited Physical Exam and Anthropometrics					X	X	X	X	X	X	X	X	X	X	X	X	X	X	X	X	X	X	X	X	X
Cord Blood and Placenta				X																					
Questionnaires					X	X	X	X	X	X	X	X	X	X	X	X	X	X	X	X	X	X	X	X	X
Labs (Blood)								X				X*		X*				X*				X*			
Labs (Urine)												X*		X*				X*				X*			
DNA/RNA								X				X*		X*				X*				X*			
Stool for microbiome				X	X	X	X	X	X	X		X*		X*				X*				X*			
OGTT																		X*				X*			
Physical activity monitor wear																		X*				X*			
Transient elastography of liver												X*		X*				X*				X*			

*Measures occur at only one visit during timeframe

### Study objectives

The primary objective is to investigate the associations of maternal risk factors including pre-pregnancy body mass index (BMI), gestational weight gain, and glycemia with offspring’s birth weight, and weight and adiposity during infancy (0–24 months of age).

Secondary objectives of the ETCHED study include:

- Studying the associations of maternal risk factors (pre-pregnancy BMI, gestational weight gain, and glycemia) with weight, adiposity, and physical growth patterns during childhood and adolescence (2–18 years of age).
- Studying the role of maternal clinical, genetic, biochemical, lifestyle, and psychosocial factors during pregnancy in mediating the associations between maternal risk factors and birth weight of the offspring, and weight, adiposity metabolic profile, and neurodevelopment during infancy, childhood, and adolescence.
- Examining the role of family, socioeconomic, and environmental factors influencing child growth patterns, body composition and neurodevelopment in infancy and childhood.
- To assess the frequency of hepatic steatosis in children and its association with BMI and biochemical alterations.
- Examining the relationship between maternal risk factors, early childhood exposures, and changes in gut microbiome during infancy, childhood, and adolescence.
- Studying the associations of maternal risk factors, including pre-pregnancy BMI, gestational weight gain, and glycemia and placental function, methylome, transcriptome, proteome and metabolome with weight, adiposity, and physical growth patters during childhood and adolescence (2–18 years of age).

### Study outcomes and data collection

The primary outcomes are birth weight, and weight, length, and age/sex adjusted BMI percentile and z-scores at 6–12 weeks, 4, 6, 12, 18, and 24-months. Secondary outcomes include BMI percentile and z-scores from 2 to 18 years of age, stool microbiome (microbial taxonomies, microbial pathways), hepatic steatosis and fibrosis, neurocognitive development, assessments of environmental and psychosocial status and biochemical measures for glycemia, lipids, renal, and liver function.

Data are collected either by in-person assessment, by review of medical records, by phone or other virtual media or a combination following the schedule of activities in Table 1. Data collected on mothers include demographics, anthropometric measures, socioeconomic and family environment history, medical history, obstetric history, laboratory evaluations, and biospecimens (blood, urine, stool, cord blood, placental tissue, and breast milk). Data collected on offspring include demographics, birth history, socioeconomic history such as living situation, childcare arrangements, parental education and employment, food insecurity, cultural practices during pregnancy, medical history, assessments of lifestyle habits such as eating and activity patterns, sleep, perceived stress, laboratory evaluations, and biospecimens (blood, urine, and stool). DNA and RNA data are collected on both mothers and offspring. The data collected in ETCHED are summarized in Table 2. Some of the information related to medical history of mothers and/or offspring is obtained by direct interview, by review of medical records, or both.

**Table 2:** Data collected in ETCHED

Participant	Type	Collected data
Mother	Demographic, socioeconomic and family environment history	• Age, race/ethnicity, marital status, approximate age of child’s father, father’s race ethnicity, father’s diabetes status • Highest level of education of parents, family size, family employment history, family income, family ownership, family hardships, household characteristics • Childcare arrangements
	Medical record review	• Clinical information, laboratory and radiological information • This includes demographic, anthropometric, clinical history, results of imaging studies, and biochemical data (will focus on periodic measures related to glycemia).
	Medical history	• Underlying health conditions, new illnesses or hospitalizations between visits, maternal history of COVID-19 infection
	Obstetric history	• Pre-pregnancy weight, delivery date(s), APGAR score, sex, weight, length, gestational age, prenatal and perinatal history, pregnancy outcomes
	Laboratory evaluations	• From blood: blood complete blood count, HbA1c, fasting glucose and insulin, adiponectin, TNF, IL-6, folate, CRP, and cytokines, biological markers for obesity and metabolic dysfunction, liver function tests, serum cholesterol, triglycerides, HDL and LDL cholesterol, apolipoproteins • Oral glucose tolerance test in the research clinic postpartum
	Biospecimens	• Blood for DNA, RNA • Stool for microbiome analysis • Breastmilk
	Mother’s procedures	• Physical exam [height, weight, waist circumference (non-pregnant) and blood pressure measurement] • Questionnaires: Unmet Social Support, Modifiable Activity Questionnaire, Pittsburg Sleep Quality Index, Adverse Childhood Experience, BLOCK-2000, Food Insecurity Questionnaire, Eating Behaviors (EARLY), Cohen’s perceived Stress Scale, Short Acculturation Scale for Hispanics (SASH) if appropriate, and cultural consideration survey
Infant	Birth history (by medical record review)	• Neonatal history such as mode of delivery, birth, weight, height, and head circumference, gestational age, APGARS, neonatal/perinatal complications, congenital anomalies
	Medical history	• Mother will be asked about: vaccination history, history of illness, including COVID diagnosis, antibiotics use, hospitalizations, ED visits, any new diagnosis.
	Medical record review	• Clinical, laboratory, and radiological information
	Laboratory evaluation	• Blood will be drawn for CBC, HbA1c, insulin, and lipids
	Biospecimens	• Cord blood for Hba1c, insulin, biomarkers such as c-peptide, leptin, adiponectin, TNF-alpha; other novel metabolomics, genetic and epigenetic tests. SARS-COVID 19 antibody • Placenta tissue for subsequent analysis of placenta function, methylome, transcriptome, proteome, metabolome, histopathology and histochemistry • Blood draw for DNA and RNA • Meconium and stool for microbiome analysis
	Procedures	• Physical exam: length, weight, head circumference • Congenital anomalies evident on clinical exam • Questionnaires that the mother will complete on behalf of the infant: cultural feeding practices, Infant consumption of food frequency, Infant feeding styles, Infant sedentary behavior, Breastfeeding formula feeding, Parent’s evaluation of development Status (PEDS) questionnaire, and Modified Checklist for Autism in Toddlers, Revised (M-CHAT-R), BLOCK Kids 2–7 Food Frequency questionnaire
Child	Medical history	• Vaccination history, history of intermittent illness, included COVID diagnosis, antibiotics use, hospitalizations, ED visits, new diagnosis
	Medical record review	• History of inter-current illness, antibiotics use, ED visits, hospitalizations, surgeries, other missed visits information such as anthropometric measures, blood pressure, lab results of tests that are part of a research and developmental milestones
	Laboratory measures	• Blood will be drawn for fasting glucose, CBC, HbA1c, OGTT (> 10 years), insulin, lipids, AST, ALT, bilirubin, cytokines and other biological markers for obesity and metabolic dysfunction • Urine will be collected for urine dip analysis and urine ACR
	Biospecimens	• Blood for DNA/RNA • Stool for microbiome analysis
	Procedures	• Physical exam: height, weight, head circumference (up to 36 months old), waist circumference, limited physical exam including Tanner staging • Questionnaires: completed by mother or guardian on behalf of the child until child is able to answer the questions in the survey independently or with parent’s help: PEDS, Netherlands Physical Activity Questionnaire, MAQ Adolescents Activity Questionnaire, BLOCK Kids diet questionnaires • Transient elastography (hepatic steatosis) in children 4 years or older: will obtain liver stiffness measurement (LS) and controlled attenuation power (CAP) measure • Accelerometer: will measure physical activity over a 7 day period, except during periods of bathing or swimming • Oral glucose tolerance test performed when children are > = 10 years of age: will collect FPG and 2-hr PG

### Sample size and power calculation

Table 3 shows the power to detect correlations of at least a given magnitude at *p* < 0.01 for recruited sample sizes of 500, 750, or 1000 paired observations (mother-child pairs, calculated with adjustment assuming 10% attrition). An alpha level of 0.01 was selected because four primary analyses are planned to correlate maternal glycemia, serum lipids, inflammatory markers, and kidney function with each primary outcome (offspring birthweight and body size up to 24 months of age). A target sample size of 750 maternal-offspring pairs is proposed, as this will provide ~ 90% power to detect a correlation coefficient of 0.15, which we take to be biologically meaningful (Table 3). Genome-wide genetic and epigenetic studies (and other studies in which large numbers of exposure variables are measured simultaneously) will require more stringent control of type I error to account for multiple testing; typically, this requires *P* < 10^− 7^ [18]. With such stringency, effect sizes will need to be somewhat larger than in standard analyses for reliable detection; with the target sample size of 750, the minimum detectable correlation at *P* < 10^− 7^ with 80% power is 0.23. We will use the false discovery rate procedure to control the type I error rate [19].

**Table 3:** Sample size and power calculations

	**Pearson correlation coefficient**		
	0.10	0.15	0.20
**Sample size**		**Power**	
500* (450)	0.35	0.76	0.96
750* (675)	0.54	0.92	> 0.99
1000* (900)	0.69	0.98	> 0.99

*Adjusted sample size accounting for 10% attrition

### Statistical analysis

The major analyses will be estimation of correlation coefficients between continuous variables, such as the correlations between maternal glycemia and offspring body size. To examine the roles of potential mediators on the relationship between maternal BMI, weight gain and glycemia with offspring body size, adiposity and metabolic outcomes, a series of linear regression models will be used in accordance with the coefficients mediation method described by MacKinnon et al. [20]. Analyses of the effect of maternal epigenetic or transcriptional changes in the serial samples will be performed using similar methods. Additional methods may also be used because statistical methods for mediation analysis are evolving.

### **Analysis of the** primary outcomes

As described above, to examine associations among maternal risk measures, and offspring weight and body size, scatterplots and grouped box plots will be constructed to examine assumptions of linearity, symmetry, and homoscedasticity. To model changes in childhood BMI over time, we will use mixed effects models to fit a piecewise growth curve model which predicts childhood BMI as a function of time.

### Sub-group analyses

Because virtually all participants (or their offspring) will be of at least part AI or Hispanic heritage, we plan to analyze subgroups defined by race and ethnicity.

### Statistical methods in handling missing data

Information pertinent to reason for missing data will be collected. Analyses will be conducted to determine whether dropout rates are associated with any key covariates. Most standard analysis methods proceed under the assumption that missing data occur at random. The analyses of factors associated with dropout rates will be used to judge the extent to which this assumption is reasonable. Multiple imputation will also be used to handle missing data. If necessary, sensitivity analyses will be conducted to determine robustness of missing data assumptions.

### Ethics approval and community outreach

The ETCHED study was approved by the National Institutes of Health’s Institutional Review Board (NIH IRB). The NIH IRB approved protocols are compliant with the U.S. Department of Health and Human Services Federal Policy for the Protection of Human Subjects (the Common Rule 2018; https://www.hhs.gov/ohrp/regulations-and-policy/regulations/45-cfr-46/revised-common-rule-regulatory-text/index.html). NIH IRB approved studies are also required to abide by the Declaration of Helsinki, and Belmont Report https://www.hhs.gov/ohrp/regulations-and-policy/belmont-report/index.html that outline the ethical principles and guideline for the protection of human subjects participating in research.

Prior to study initiation, extensive local and community outreach was done by the Principal Investigator and the study team in both Hispanic and AI/AN communities. In addition, for inclusion of AI/AN study participants in ETCHED, appropriate consultations with Tribal partners were undertaken as directed by the NIH IRB and NIH’s Tribal Health Research Office (THRO). In addition, the study design and feasibility issues were discussed with staff and physicians at VHMC, and relevant suggestions were integrated into the protocol. Although ETCHED is a longitudinal observational study, it benefited from the valuable experience gained by PECRB investigators and staff working with prenatal participants and mother/child dyads in the prior clinical trials that were conducted in this population. The data collected in the ETCHED study are uniform across both Hispanic and AI/AN ethnic/racial group, informed consents are obtained from the participants prior to any study procedure, and assent will be obtained from child participants in the future.

### Dissemination

The results of the analyses for primary and secondary outcomes will be presented at local, national, and international conferences. The findings will be published in articles in English in relevant peer-reviewed scientific journals. In addition, any new study findings will be shared with the study participants and the healthcare providers in the Phoenix Metropolitan area. Since our study participants are from Hispanic and various AI/AN communities, additional dissemination of study findings within local Indigenous American Communities will be offered in consultation with NIH and the THRO. This may include presentation of novel study findings to local AI/AN and Hispanic communities and institutions in the southwestern US that are engaged in improving the health in minority and vulnerable populations.

## Discussion

ETCHED is a life course study that will examine biological and environmental risk factors for obesity and cardiometabolic risk from the intrauterine period to early childhood and adolescence. The study population comprising Hispanic and American Indian/Alaska Native maternal/child dyads, were selected since they are at high-risk of obesity and type 2 diabetes compared to other racial/ethnic groups in the United States and planning and development of the protocol incorporated input from individuals in the target population. The steady funding support from the National Institutes of Health’s Intramural Research Program will ensure consistency and uniformity in implementing study procedures and data collection during the planned study duration of 23-years in this ongoing study. Given that obesity is a multifactorial complex disease, mechanistic insight is needed to predict which individual more precisely may be at risk for obesity and cardiometabolic disease and identify targets for intervention. To address this knowledge gap and improve understanding of early life obesity and associated metabolic risk, we will also conduct a unique phenotypic multi-omics based analytical approach utilizing clinical data, metabolomics, proteomics, and microbiomics, to investigate alterations at a molecular level and build a novel prediction model of those factors that may ultimately lead to obesity. Limitations include potential for loss to follow up and attrition over time, particularly in a vulnerable inner-city population. The large amount of data and biological samples collected longitudinally will provide a unique opportunity for a robust epidemiological assessment and additional integrated omics-based data analysis.

## Data Availability

No datasets were generated or analysed during the current study.

## Abbreviations

US: United States
AI/AN: American Indian/Alaska Native
T2D: Type 2 diabetes
T1D: Type 1 diabetes
DOHaD: Developmental Origins of Health and Disease
ETCHED: Early Tracking of Childhood Health Determinants
NIDDK: National Institute of Diabetes and Digestive and Kidney Diseases
PECRB: Phoenix Epidemiology and Clinical Research Branch
VHMC: Valleywise Health Medical Center
BMI: Body mass index
THRO: Tribal Health Research Office

## References

[1] Cheryl D, Fryar MD, Carroll J, Afful S, DoHaNE. Prevalence of overweight, obesity, and severe obesity among children and adolescents aged 2–19 years: United States, 1963–1965 through 2017–2018. In. Edited by Statistics NCfH. CDC 2021.

[2] Stierman B, Afful J, Carroll MD, Chen T-C, Davy O, Fink S, Fryar CD, Gu Q, Hales CM, Hughes JP et al. National health and nutrition examination survey 2017–March 2020 Prepandemic data files -- Development of files and prevalence estimates for selected health outcomes. In. Hyattsville, MD: 10.15620/cdc:106273; 2021. 10.15620/cdc:106273 PMC11513744 39380201

[3] Bullock A, Sheff K, Moore K, Manson S. Obesity and overweight in American Indian and Alaska Native children, 2006–2015. Am J Public Health. 2017;107(9):1502–7. 28727519 10.2105/AJPH.2017.303904 PMC5551606

[4] Ogden CL, Fryar CD, Martin CB, Freedman DS, Carroll MD, Gu Q, Hales CM. Trends in obesity prevalence by race and hispanic origin-1999-2000 to 2017–2018. JAMA. 2020;324(12):1208–10. 32857101 10.1001/jama.2020.14590 PMC7455882

[5] Perng W, Conway R, Mayer-Davis E, Dabelea D. Youth-Onset type 2 diabetes: the epidemiology of an awakening epidemic. Diabetes Care. 2023;46(3):490–9. 36812420 10.2337/dci22-0046 PMC10090267

[6] Mayer-Davis EJ, Lawrence JM, Dabelea D, Divers J, Isom S, Dolan L, Imperatore G, Linder B, Marcovina S, Pettitt DJ, et al. Incidence trends of type 1 and type 2 diabetes among youths, 2002–2012. N Engl J Med. 2017;376(15):1419–29. 28402773 10.1056/NEJMoa1610187 PMC5592722

[7] Tanamas SK, Reddy SP, Chambers MA, Clark EJ, Dunnigan DL, Hanson RL, Nelson RG, Knowler WC, Sinha M. Effect of severe obesity in childhood and adolescence on risk of type 2 diabetes in youth and early adulthood in an American Indian population. Pediatr Diabetes. 2018;19(4):622–9. 29282818 10.1111/pedi.12627

[8] Nelson RG, Newman JM, Knowler WC, Sievers ML, Kunzelman CL, Pettitt DJ, Moffett CD, Teutsch SM, Bennett PH. Incidence of end-stage renal disease in type 2 (non-insulin-dependent) diabetes mellitus in Pima indians. Diabetologia. 1988;31(10):730–6. 3240833 10.1007/BF00274774

[9] Franks PW, Hanson RL, Knowler WC, Sievers ML, Bennett PH, Looker HC. Childhood obesity, other cardiovascular risk factors, and premature death. N Engl J Med. 2010;362(6):485–93. 20147714 10.1056/NEJMoa0904130 PMC2958822

[10] Pettitt DJ, Aleck KA, Baird HR, Carraher MJ, Bennett PH, Knowler WC. Congenital susceptibility to NIDDM. Role of intrauterine environment. Diabetes. 1988;37(5):622–8. 3360218 10.2337/diab.37.5.622

[11] Dabelea D, Hanson RL, Lindsay RS, Pettitt DJ, Imperatore G, Gabir MM, Roumain J, Bennett PH, Knowler WC. Intrauterine exposure to diabetes conveys risks for type 2 diabetes and obesity: a study of discordant sibships. Diabetes. 2000;49(12):2208–11. 11118027 10.2337/diabetes.49.12.2208

[12] Page KA, Romero A, Buchanan TA, Xiang AH. Gestational diabetes mellitus, maternal obesity, and adiposity in offspring. J Pediatr. 2014;164(4):807–10. 24388326 10.1016/j.jpeds.2013.11.063 PMC3962700

[13] Knowler WC, Pettitt DJ, Saad MF, Bennett PH. Diabetes mellitus in the Pima indians: incidence, risk factors and pathogenesis. Diabetes Metab Rev. 1990;6(1):1–27. 2192853 10.1002/dmr.5610060101

[14] Pettitt DJ, Jovanovic L. The vicious cycle of diabetes and pregnancy. Curr Diab Rep. 2007;7(4):295–7. 17686406 10.1007/s11892-007-0047-x

[15] Barker DJ, Winter PD, Osmond C, Margetts B, Simmonds SJ. Weight in infancy and death from ischaemic heart disease. Lancet. 1989;2(8663):577–80. 2570282 10.1016/s0140-6736(89)90710-1

[16] Hales CN, Barker DJ. Type 2 (non-insulin-dependent) diabetes mellitus: the thrifty phenotype hypothesis. 1992. Int J Epidemiol. 2013;42(5):1215–22. 24159065 10.1093/ije/dyt133

[17] Gillman MW. Developmental origins of health and disease. N Engl J Med. 2005;353(17):1848–50. 16251542 10.1056/NEJMe058187 PMC1488726

[18] Benjamini Y, Hochberg Y. Controlling the false Discovery rate: a practical and powerful approach to multiple testing. J Royal Stat Soc Ser B (Methodological). 1995;57(1):289–300.

[19] Rakyan VK, Down TA, Balding DJ, Beck S. Epigenome-wide association studies for common human diseases. Nat Rev Genet. 2011;12(8):529–41. 21747404 10.1038/nrg3000 PMC3508712

[20] MacKinnon DP, Fairchild AJ, Fritz MS. Mediation analysis. Annu Rev Psychol. 2007;58:593–614. 16968208 10.1146/annurev.psych.58.110405.085542 PMC2819368

